# Supplementary material for: LIX1 Controls MAPK Signaling Reactivation and Contributes to GIST-T1 Cell Resistance to Imatinib
Source: Int J Mol Sci. 2023 Apr 12;24(8):7138. doi: 10.3390/ijms24087138 (PMC10138740; doi:10.3390/ijms24087138)
Supplement: Supplementary file 1 [file ijms-24-07138-s001.zip › ijms-2308971-supplementary.pdf]

## Supplementary Table 1: List of Antibodies.

-Polyclonal rabbit anti-AKT antibody (Cell Signaling Technology Cat# 9272, RRID:AB_329827)

-Monoclonal rabbit anti-phosphorylated AKT (Ser473) antibody (Cell Signaling Technology Cat# 4060, RRID:AB_2315049)

-Monoclonal mouse anti-ERK1/2 (p44/42) antibody (Cell Signaling Technology Cat# 4696, RRID: AB_390780)

-Monoclonal rabbit anti-phosphorylated p44:42 MAPK (ERK1/2) (Thr202/Tyr204) antibody (Cell Signaling Technology Cat# 4376, RRID:AB_331772)

-Monoclonal mouse anti-GAPDH (p44/42) antibody (Sigma Cat# G8795, RRID: AB_1078991)

-Monoclonal rabbit anti-KIT antibody (DB Biotech Cat# DB 062, RRID:AB_2315699)

-Monoclonal rabbit anti-phosphorylated KIT (Tyr703) antibody (Cell Signaling Technology Cat# 3073, RRID:AB_1147635)

-Polyclonal rabbit anti-LIX1 antibody (Aviva Systems Biology Cat# ARP53147_P050, RRID:AB_2045922)

-Monoclonal rabbit anti-TAZ antibody (Cell Signaling Technology Cat# 72804, RRID:AB_2904134)

-Monoclonal rabbit anti-YAP1 antibody (Cell Signaling Technology Cat# 14074, RRID: AB_2650491)

**Supplementary Table 2: Human gene-specific primers used for RT-qPCR.**

| Target | Forward primer (5'-3') | Reverse primer (5'-3') | Amplicon (bp) |
|---|---|---|---|
| ***HMBS*** | GGC AAT GCG GCT GCA A | GGG TAC CCA CGC GAA TCA C | 64 |
| ***LIX1*** | CTG CAG TGA CCC TGA CAC TT | AAT CTG GCC TCT GCC ATC AC | 118 |
| ***SPROUTY2*** | ATG GCA TAA TCC GGG TGC AA | ACT TGC CAC AGT CCT CAC AC | 92 |
| ***SPROUTY4*** | CCT TCA TGG GTG CTC TCT CC | GCT TTG CAG ATG ACG CTG TT | 148 |
| ***YWHAZ*** | ACT TTT GGT ACA TTG TGG CTT CAA | CCG CCA GGA CAA ACC AGT AT | 94 |
